# Big Data: A Parallel Particle Swarm Optimization-Back-Propagation Neural Network Algorithm Based on MapReduce

**DOI:** 10.1371/journal.pone.0157551

**Published:** 2016-06-15

**Authors:** Jianfang Cao, Hongyan Cui, Hao Shi, Lijuan Jiao

**Affiliations:** 1 Computer Science and Technology Department, Xinzhou Teachers University, Xinzhou, China; 2 College of Computer Science and Technology, Taiyuan University of Science and Technology, Taiyuan, China; Tianjin University, CHINA

## Abstract

A back-propagation (BP) neural network can solve complicated random nonlinear mapping problems; therefore, it can be applied to a wide range of problems. However, as the sample size increases, the time required to train BP neural networks becomes lengthy. Moreover, the classification accuracy decreases as well. To improve the classification accuracy and runtime efficiency of the BP neural network algorithm, we proposed a parallel design and realization method for a particle swarm optimization (PSO)-optimized BP neural network based on MapReduce on the Hadoop platform using both the PSO algorithm and a parallel design. The PSO algorithm was used to optimize the BP neural network’s initial weights and thresholds and improve the accuracy of the classification algorithm. The MapReduce parallel programming model was utilized to achieve parallel processing of the BP algorithm, thereby solving the problems of hardware and communication overhead when the BP neural network addresses big data. Datasets on 5 different scales were constructed using the scene image library from the SUN Database. The classification accuracy of the parallel PSO-BP neural network algorithm is approximately 92%, and the system efficiency is approximately 0.85, which presents obvious advantages when processing big data. The algorithm proposed in this study demonstrated both higher classification accuracy and improved time efficiency, which represents a significant improvement obtained from applying parallel processing to an intelligent algorithm on big data.

## Introduction

A Back-Propagation (BP) neural network is a type of multi-layered feed-forward neural network that learns by constantly modifying both the connection weights between the neurons in each layer and the neuron thresholds to make the network output continuously approximate the desired output [1]. Because a BP neural network is robust and can realize any complex nonlinear mapping relation, this technique has been widely used in many fields [2]. In terms of speed prediction, to better utilize wind power, Guo et al. [3] constructed a hybrid wind speed prediction model using a BP neural network that eliminates seasonal effects from actual wind speed datasets using a seasonal exponential adjustment. They conducted a case study in Minqin County, Gansu Province that satisfactorily eliminated the seasonal effects from the prediction results. Xu et al. [4] applied a BP neural network to debris flow control engineering and proposed a method for predicting the average velocity of debris flow. In the medical field, Zhang et al. [5] used a BP neural network to construct an m-order nonlinear model to describe the complex relationships between surface electromyogram signals and the joint angles of a human leg. The model inputs were preprocessed sEMG time series, and the outputs were the hip, knee and ankle joint angles, with an average angle estimation root mean square error of less than 5° for spinal cord injury patients. Cheng et al. [6] proposed a new method based on a BP neural network to predict facial deformation following a complete denture prosthesis. By analyzing the relationship between one third of the facial area and the completed denture, this method accurately predicted deformation of the facial soft tissue. To solve cotton pest problems in agricultural fields, Zhang et al. [7] designed a cotton disease and pest damage identification method based on rough sets and a BP neural network that accurately identified 4 cotton diseases. In digital image processing, Pan et al. [8] used a BP neural network to conduct automatic recognition of woven fabric patterns by extracting fabric texture features with a black and white co-occurrence matrix. In other areas, Hu [9] proposed a type of intrusion detection algorithm that used a BP neural network to solve the relatively high false negative rate and high false alarm rate problems with traditional intrusion detection algorithms. Liu et al. [10] applied a BP neural network to establish a forecasting model for port throughput and achieved good results when forecasting the port throughput in 2011 and 2012. However, the BP neural network algorithm is based on the idea of an error gradient descent function; thus, it does not have global search capability. In addition, the connection weights between the layers and the neuron’s thresholds, which can take on random values between 0 and 1 in the initial training stage, lead to slow convergence by the algorithm and do not necessarily lead to an optimal solution [11]. Later, researchers gradually introduced some metaheuristic optimization algorithms. Wang et al. [12–14] presented an improved firefly algorithm, a biogeography-based krill herd algorithm and a chaotic krill herd algorithm to solve complex optimization tasks. Chiroma H. et al. [15] discussed the applications of a bio-inspired flower pollination algorithm, which motivated other researchers in the bio-inspired algorithms research community to further improve the effectiveness of the flower pollination algorithm as well as to apply the algorithm in other fields to solve optimization problems in engineering and industry. Saadi et al. [16] proposed a new metaheuristic algorithm called Ringed Seal Search that found the optimal solution. This algorithm searched and selected the best lair by performing a random walk to find new lairs. In recent years, optimization algorithms have usually been used to optimize the weights of neural networks and solve practical problems in various fields. Nawi et al. [17] used the PSO algorithm to optimize the weights of recurrent neural networks and conduct data classification. Chiroma et al. [18] applied an artificial neural network optimized by the PSO algorithm to predict OPEC CO_2_ emissions. Chiroma H. et al. [19, 20] predicted crude oil prices using a neural network optimized by a genetic algorithm. The optimization and application of the initial weights and thresholds of the BP neural network have also been studied intensively. The most widely used optimization algorithms are the genetic algorithm (GA) and the particle swarm optimization (PSO) algorithm. Ding et al. [21] combined a GA with BP neural networks to accelerate the training of the BP network—which to a certain extent overcomes the disadvantages of the BP becoming easily stuck in a local minimum—and performed an experimental analysis of the UCI dataset. Yu et al. [22] also used a genetic algorithm to optimize a BP neural network. They improved the additional momentum factor and self-adaptive learning rate and established a natural gas load forecasting model to make short-term forecasts of natural gas loads in Shanghai. Gao et al. [23] used the GA to optimize the initial weights and thresholds of a BP neural network and was able to predict housing prices in Guiyang City with improved accuracy. Wang et al. [24] applied a PSO-optimized BP neural network algorithm to estimate the cost of plastic injection molding parts, improving both the convergence rate of the algorithm and the cost estimation accuracy rate. Similar to [3], Ren et al. [25] also predicted wind speed; however, in contrast to [3], Ren et al. first used a PSO algorithm to optimize the initial thresholds and weights of the BP neural network and then established a forecasting model that obtains improved accuracy compared to [3]. In addition, various other algorithms have been combined with the BP neural network to improve its performance. To improve classification results, Jia et al. [26] proposed a type of PLS-HCA-BP algorithm that uses partial least squares (PLS) to reduce the feature dimensions of samples, which simplifies the network structure and improves the speed of convergence in hierarchical cluster analysis (HCA), therein obtaining favorable experimental results. Wang et al. [27] established a target threat estimation model using Glowworm Swarm Optimization and the BP neural network. The above-mentioned studies, combined with other algorithms, have optimized BP neural networks and improved their performance. Compared with standalone BP neural networks, such combinations have achieved good results. However, regardless of the optimization employed, BP neural networks always show good performance when the sample size is small; however, when the sample set size increases, the time efficiency of these algorithms sharply and intolerably declines.

With the arrival of the big data era, sample datasets are becoming increasingly large. A bottleneck problem is being faced in terms of hardware capabilities for the above-mentioned traditional serial algorithm form of the BP neural network. Moreover, the training time for the algorithm becomes very lengthy. As a result, the efficiency of the system decreases significantly. Scholars are beginning to study parallel designs for traditional algorithms. Feng et al. [28] achieved a parallel form of an algorithm for a BP neural network on the Sunway Blue Light Supercomputer based on MPI techniques and made predictions of traffic flow. Zheng et al. [29] designed a type of multi-BP neural network parallel integration model and performed multi-semantic image classifications, therein obtaining good experimental results under the MPI Cluster environment. Liu [30] conducted parallel processing of a large-scale matrix operation in the PVM parallel environment, effectively reducing the time required for matrix operations. Guo et al. [31] designed a parallel BP neural network based on a field programmable gate array (FPGA) to address big data. Wang et al. [32] implemented an efficient GPU to train large-scale recurrent neural networks and achieved a good experimental result. However, the parallel design (based on MPI, PVM, FPGA and GPUs) requires a clear understanding of the computer hardware architecture by the developers, and the communication between nodes is time consuming. These factors make such systems difficult to build and apply [33].

The MapReduce framework is based on Hadoop and has become popular in recent years. MapReduce is a type of parallel computing model oriented toward distributed environments. This model provides developers with a complete programming interface, does not require them to understand the computer architecture, and has gradually become a research hotspot for current studies on parallel algorithm design [34]. Therefore, researchers have proposed some algorithms for processing big data. Kim et al. [35] used a MapReduce model to design a density-based clustering algorithm suitable for processing big data and achieved good experimental results. Attribute reduction is an important research question in rough set theory. When addressing big data, the traditional attribute reduction algorithms appear to be inadequate. Jin et al. [36] proposed a hierarchical attribute reduction algorithm that executed in parallel using MapReduce on both data and tasks, therein saving a great deal of time. Scardapane et al. [37] proposed a decentralized training algorithm for Echo State Networks in distributed big data applications. To fulfill the potential of artificial neural networks for big data applications, Liu et al. [38] used the MapReduce framework to implement parallelized neural networks and solve existing problems faced by traditional artificial neural networks. Simultaneously, the applications of big data have gradually increased in various fields, especially in the biomedical fields. Zou et al. [39] applied the MapReduce framework to bioinformatics and used it to conduct next-generation sequencing. Subsequently, Zou et al. [40] used the MapReduce model to solve bottleneck problems in massive multiple sequence alignments (MSAs) of homologous DNA and genome sequences. Shen et al. [41] proposed a quantitative quality control method for radiotherapy and chemotherapy based on an artificial neural network to process medical big data. Chung et al. [42] used a parallel deep neural network to train big data on Blue Gene. Elsebakhi et al. [43] adopted functional networks based on propensity scores and Newton-Raphson maximum-likelihood optimizations as a new large-scale machine learning classifier to improve the classification performance when faced with biomedical big data. In other areas, to address increasing traffic flow data efficiently, Cheng et al. [44] proposed a prediction method to process distributed traffic flow using a parallel programming model based on MapReduce. Gu et al. [45] designed a parallel computing platform to make instant messaging deliveries. Stateczny et al. [46] solved the reduction problems of hydrographic big data. These studies, using MapReduce parallel processing, all do a good job of improving the time efficiency of their respective systems. Applications based on MapReduce are gradually increasing. However, there are few studies concerning the PSO algorithm or that investigate MapReduce distributed parallel processing of BP neural networks and their application to the semantic classification of digital images.

To solve the above-mentioned problems, this study proposes a parallel PSO-BP neural network algorithm and applies it to the classification of scene images. The parallel processing mechanism of this algorithm is based on the MapReduce parallel programming model and uses the PSO algorithm to optimize the initial weights and thresholds of the BP neural network. Then, different multiple optimized parallel BP neural networks are used to train different sample sets, which not only ensures that the BP neural network can obtain the optimal solution but also accelerates the convergence rate. This approach effectively reduces the impact of sample diversity and complexity on the performance of the BP neural network and shortens the required training time.

## PSO-BP Neural Network Algorithm

The PSO algorithm, when introduced into a BP neural network to optimize its initial weights and thresholds, is well suited for addressing some of the deficiencies caused by the randomness of the initial weights and thresholds of BP neural networks [24].

### PSO-optimized BP neural network model

**Fig 1:**
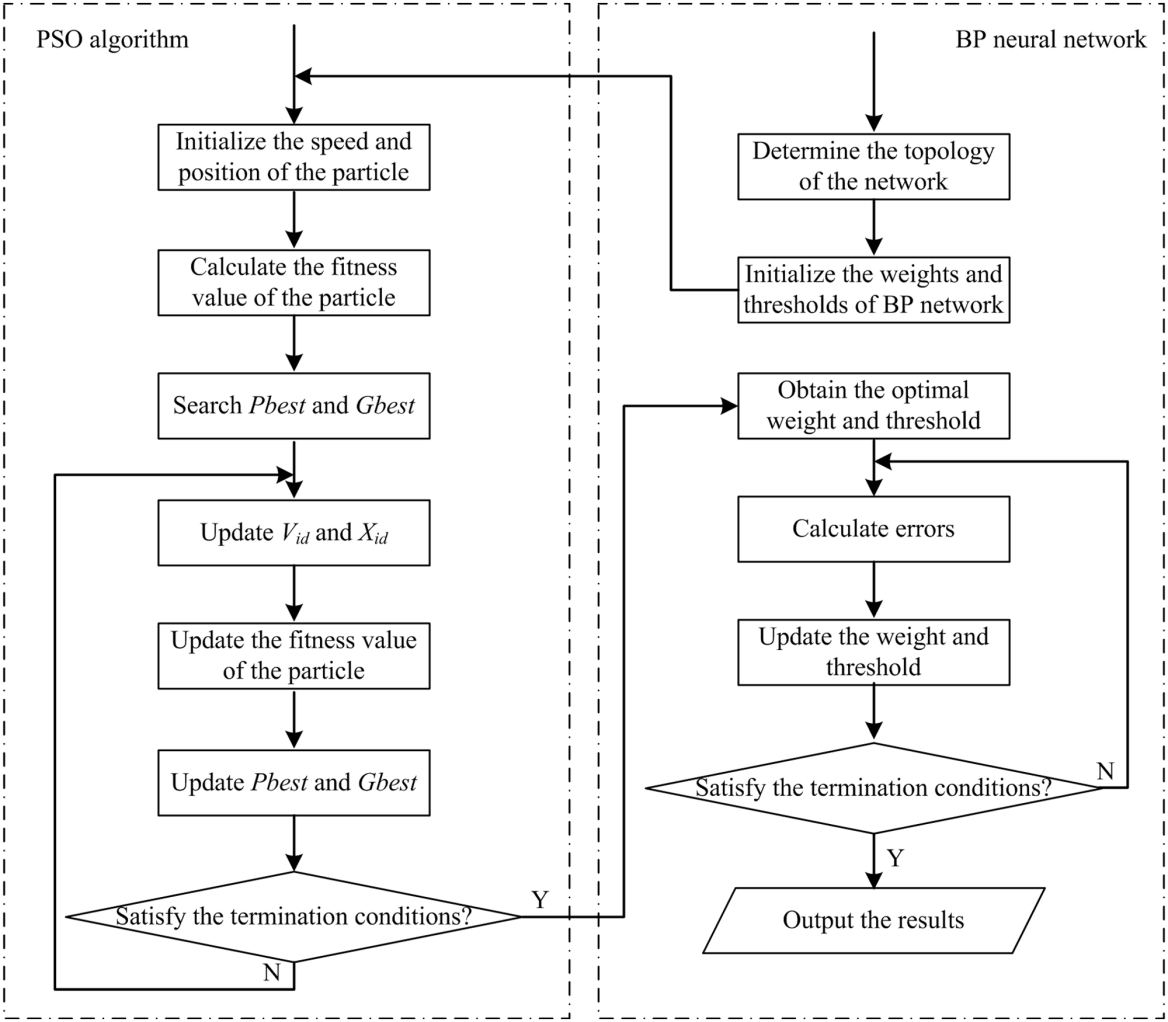
PSO-BP neural network algorithm model.

The basic idea behind using the PSO algorithm to optimize the BP neural network is to combine them, using the initial connection weights between BP neural network layers and the initial thresholds between neural nodes, to optimize the distribution, execute global searches within the solution space, and find the optimal initial weights and thresholds of the BP neural network at a rapid convergence rate. Subsequently, the initial weights and thresholds obtained by the BP neural network can be used for training and testing the sample set. Fig 1 shows a flowchart of this optimized model.

### Steps in the PSO-optimized BP neural network algorithm

The main steps of the algorithm are as follows:

Optimization phase of PSO algorithm:

1. Initialize PSO parameters (population size, speed and position of particles and iterations).
2. Determine the structure of the BP neural network and generate population particles.
  Particles: *X*_*i*_ = (*x*_*i*1_,*x*_*i*2_,_…_.*x*_*iD*_)^*T*^, *i* = 1,2,…,*n*
  $$D=RD_{1}+D_{1}D_{2}+D_{1}+D_{2}$$ (1)
  where R, *D*_1_ and *D*_2_ represent the number of nodes in the input layer, hidden layer and output layer of the BP neural network, respectively.
3. Design the fitness function. The BP neural network trains on the training samples in accordance with Particle *X*_*i*_ in Eq (2) to set weights and thresholds until it generates the expected output $\bar{y_{i}}$ The fitness function of the individual, *X_i_*, is defined as
  $$fit_{i}=\sum_{j=1}^{M-1}(\bar{y_{j}}-y_{j}{)}^{2}\;(i=1,2,\cdots ,n)$$ (2)
4. Calculate each particle’s fitness value and evaluate Population *X*. Calculate the fitness value of each individual particle *X*_*i*_.
5. Update the individual’s optimal fitness value *Pbest* and the particles’ positions. For each particle *X*_*i*_, if its current fitness value is smaller than its optimal fitness value *Pbest*, then use the current fitness value to update the optimal fitness value *Pbest* and the position of particle *X*_*i*_.
6. Update the population fitness value *Gbest*. For each particle *X*_*i*_, if its fitness value is smaller than the optimal fitness value *Gbest* for the current population, then use the fitness value of the current particle to update the optimal fitness value *Gbest*.
7. Iteratively update the speed and position of particles to generate new populations. According to Formulas (3) and (4), constantly update the speed and position of each particle *X*_*i*_, as shown below:
  $$V_{id}^{k+1}=\omega V_{id}^{k}+c_{1}r_{1}(P_{id}^{k}-X_{id}^{k})+c_{2}r_{2}(P_{gd}^{k}-X_{id}^{k})\;(d\in [1,D],i\in [1,n])$$ (3)
  $$X_{id}^{k+1}=X_{id}^{k}+V_{id}^{k+1}\;(d\in [1,D],i\in [1,n])$$ (4)
  where *ω* is the inertia weight; *k* is the number of iterations; *V_id_* is the speed of Particle *X_i_*; *c*_1_ and *c*_2_ are acceleration factors, which are not smaller than zero; and *r*_1_ and *r*_2_ are arbitrary numbers between 0 and 1.
8. Judge whether to halt the iteration based on whether the optimal initial weights and thresholds of the BP neural network have been generated. The overall fitness variance (Formula (5)) is used to judge whether the PSO algorithm converges. If the overall fitness variance of the particle population is smaller than the given threshold, then the algorithm has converged. The optimal solution is output as the initial weights and thresholds. Otherwise, the iterations continue.
  $$\sigma^{2}=-\sum_{i=1}^{n}(\frac{f_{i}-f_{avg}}{f}{)}^{2}$$ (5)
  where *n* is the number of particles in the current population, *f*_*i*_ is the fitness value of particle *X*_*i*_, and *f*_*avg*_ is the average fitness of the current population particle.

The training phases of the BP neural network are as follows:

1. Initialize the network. The network structure, expected output and learning rate are determined according to the sample characteristics. The PSO algorithm optimization is used to derive the optimal individual solution for the initial weight value and threshold of the network.
2. Input the training sample and calculate the output of the network layers.
3. Calculate the learning error of the network.
4. Correct the connection weight values and thresholds of the layers.
5. Judge whether the error satisfies the expectation requirements and whether the number of iterations has reached the set training limit. If either condition is met, then the training ends. Otherwise, the iterative learning process continues.

## Parallel Implementation of the PSO-BP Neural Network Algorithm

The arrival of the big data era poses a challenge to traditional machine learning algorithms. In information technology, big data is a collection of datasets so large and complex that they become difficult to process using available database management tools or traditional data processing applications. Big data is usually composed of datasets with sizes beyond the ability of commonly used software tools, which are unable to capture, curate, manage, or process such data within a reasonable elapsed time [47]. The challenges include capture, curation, storage, search, sharing, analysis, and visualization. Therefore, both the time and space efficiency of traditional algorithms decrease dramatically when addressing big data.

Although the PSO-BP neural network algorithm improves the performance of the traditional BP neural network algorithm, as the data size increases, the BP neural network training time increases as well, eventually raising efficiency issues. The MapReduce parallel programming framework provides a type of distributed parallel calculation environment for big data processing. Faced with booming data growth in a big data environment, there is a need to improve the time efficiency and accuracy of BP neural networks. Therefore, this study's goals are to solve the low time efficiency problem and the poor data classification accuracy of traditional BP neural networks optimized by the PSO algorithm as proposed in [25]. To that end, this study conducts a parallel implementation of the PSO-BP neural network algorithm within the MapReduce framework.

### MapReduce programming model

**Fig 2:**
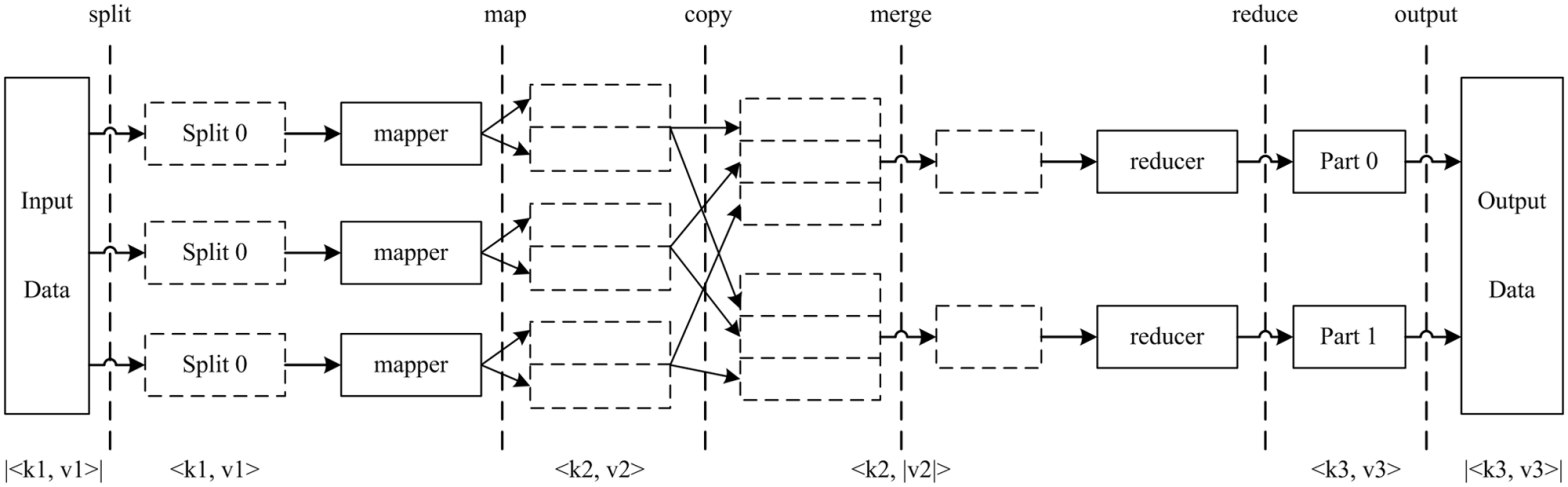
The MapReduce programming model process.

MapReduce [47] is based on Hadoop, which is a type of distributed parallel programming model developed by Google Inc. for processing big data. MapReduce separates the calculation process for data into Map and Reduce stages, which correspond to the execution of a mapper() function and a reducer() function, respectively. The process requires data to be input in the form of key-value pairs (e.g., <key, value>, where the key can be regarded as the data serial number and the value can be regarded as the data value). In the Map stage, MapReduce segments data into Splits of identical sizes and processes each Split in the form of key value pairs <*k1*, *v1*> to create the formal input. It executes the mapper() function to generate intermediate results in the form of <*k2*, *v2*>, which are ranked according to the value of *k2*. The *v2* values whose *k2* values are the same are combined to form a new list <*k2*, *list(v2)*> and, finally, grouped according to the scope of *k2* to form Reduce tasks. In the Reduce stage, the outputs of the Map tasks are integrated and ranked, <*k2*, *list(v2)*> is taken as the input, and the reduce() function is executed to obtain the key value pair <*k3*, *v3*>, which is output to the Hadoop File System (HDFS). A flowchart of the MapReduce process is shown in Fig 2.

### Parallel design and realization of PSO algorithm

#### Design and realization of PSO-Map()

Due to the data input format utilized by the MapReduce programming model, this study first converts the data input by the PSO algorithm into the <*key*, *value*> format, where *key* represents the serial number of a particle and value represents the attribute of a particle. In the Map stage—particle initialization and fitness evaluation—the optimal particles for individual populations and other operations are calculated. The Map process for the PSO algorithm is designed as follows:

Input: particle id, particle attribute value

Output: key, optimal particle set

PSO-Map(particle id, particle attribute value)

{

For each particle, obtain its value;

fit-value = fit(value); //calculate the fitness value of the updated particle

//iteratively update the position and speed of particle

if (satisfy condition)

key = new-key(key);

output key, optimal particle set;

}

#### Design and realization of PSO-Reduce()

In the Reduce stage, the optimal particle sets generated by the Map tasks are received and integrated, and the position and speed of the optimal particle set are globally updated. If the termination condition is satisfied, the optimal particle of the whole population is output. The Reduce process for the PSO algorithm is designed as follows:

Input: key, optimal particle set

Output: key, optimal particles

PSO-Reduce(key, optimal particle set)

{

Obtain the fit-value of each optimal particle in the population;

//globally iteratively update population

if (the optimal solution of the problem is obtained|| and the maximum iteration times are reached)

output key, optimal particle;

}

When the parallel iterations of the PSO algorithm are complete, the optimal particles obtained are the initial weights and thresholds for the BP neural network.

### Parallel design and realization of the BP neural network

**Fig 3:**
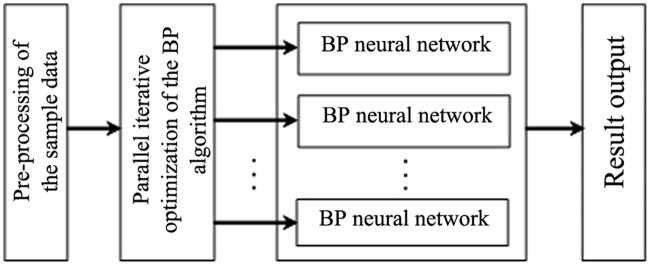
A parallel model of the BP neural network.

To overcome the shortcomings of the BP neural network, including hardware overhead and excessive training time, this study also conducts a parallel design for the BP neural network based on MapReduce, therein using Map and Reduce tasks to implement automatic parallel operations within the multi-layered BP neural network. This approach greatly reduces the training time and simultaneously improves the training precision. The model structure is shown in Fig 3.

#### Design and realization of BP-Map()

In the Map stage, based on the input, the Map tasks calculate the actual output of the network layer by layer. Then, the actual output and the expected output are compared to calculate the network learning error. Finally, based on the learning error, updated connection weight values in the network are calculated. The Map tasks for the BP neural network are designed as follows:

Input: sample id, sample feature value

Output: the corresponding weights *ω* of the sample and the amount Δ*ω* by which the weights were updated

BP-Map(Sample id, sample feature value)

{

//For each sample,

calculate the output of network layers;

and calculate the learning error of the network;

For each connection weight *w*, calculate the updated amount Δ*w* for the weight;

Output(*w*, Δ*w*);

}

#### Design and realization of the BP-Combine() function

In the MapReduce parallel programming model, the Combine() function can perform local processing of the intermediate result generated in the Map stage, thereby greatly reducing the communication overhead. As the size of the sample data that the BP neural network uses for training gradually increases, it becomes imperative to process the results generated by Map tasks using the Combine() function before using them as input in the Reduce tasks. In the parallel design process of the BP neural network, the Combine() function is designed as follows:

Input: key value pair <*w*, Δ*w>*

Output: key value pair <*w*, Δ*w>*

BP-Combine(*w*, Δ*w*)

{

Initial variable count = 0; //count the number of training samples

//resolve each training sample

and process the three-dimensional coordinate values of Δ*w*;

count←count+1;

*w*←*w*;

Collect all the key value pairs whose *w* values are the same, and conduct local reduction to derive Δ*w*;

Output *w*←*w*;

}

#### Design and realization of BP-Reduce()

In the Reduce stage, the output of the Combine() function is used as input. Next, the total number of samples whose weights are the same is calculated, and their weights are updated. The Reduce tasks for the BP neural network are designed as follows:

Input: the output of the Combine function: <*ω*′,Δ*ω*′>

Output: $<\omega^{\prime },\sum_{i=1}^{n}\Delta \omega^{\prime }/n>$

BP-Reduce (*ω*′,Δ*ω*′)

{

Add the Δ*ω*′ of the samples whose *ω*′ s are the same to obtain $\sum_{i=1}^{n}\Delta \omega^{\prime }$;

calculate the average amount with which to update every weight; and

output *ω*′,$\sum_{i=1}^{n}\Delta \omega^{\prime }/n$;

}

## Experiment and Results Analysis

To validate the performance of the parallel PSO-BP neural network algorithm proposed in this study, we tested it on a semantic classification task with a large number of scene images on the Hadoop platform.

### Experimental environment and data

The experimental environment was a Hadoop cluster composed of five computers in an intranet. One computer acted as the master node, and the other four computers acted as slave nodes. All the nodes were equipped with 4G dual-core processors, 1 TB hard disks, and the Ubuntu operating system. Configured as described, the system can obtain the desired results even when dealing with tens of thousands of images. As the size of the image database increases, additional processing power can be added by increasing the number of slave nodes in the Hadoop cluster [48].

The experimental data stemmed from the SUN Database image library (http://groups.csail.mit.edu/vision/SUN/), which consists of 131,067 images in 908 categories freely available to researchers. At present, the Sun database is available as a free big data image set. This study constructed 5 datasets (using a combination of random selection by a computer and artificial selection) and named them sequentially as “Data1” through “Data5.” Data1 contained 300 scene images in 3 categories, Data2 contained 800 scene images in 5 categories, Data3 contained 2,000 scene images in 8 categories, Data4 contained 5,000 scene images in 12 categories, and Data5 contained 15,000 scene images in 15 categories. For processing convenience, we convert all experiment images into images of 300 pixels × 300 pixels, and the size of each image is approximately 265 KB. Thus, the size of the 15,000 images used in this paper is approximately 3.98 GB.

Because the color feature is the main characteristic reflecting the implied semantics of scene images [2], in the preprocessing stage for the sample data in this study, 60-dimensional color feature vectors were selected in the HSV color space in the OpenCV environment. In this paper, 60-dimensional color feature vectors are used as the input, and the image category is the output. Therefore, for the BP neural network, after the individual PSO was conducted, the number of input nodes *n*_*i*_ was set to 60, and the number of output nodes was *n*_*o*_ to 1. The number of hidden nodes *n*_*h*_ was determined to be 8 through repeated experiments according to Formula (6). Then, the structure of an individual BP neural network was 60-8-1. Ten BP neural networks with the same structures and all optimized using the PSO algorithm were used in the study to perform parallel categorizations of the scene images.

$$n_{h}=\sqrt{n_{i}+n_{o}}+\alpha \;\alpha \in [0,1]$$ (6)

where *α* is an arbitrary decimal value between 0 and 1.

### Experimental results and analysis

To validate the performance of the algorithm proposed in this study, we conducted experimental comparisons by evaluating aspects such as classification accuracy, speedup ratio, and efficiency.

#### Classification accuracy

Under different image scales, and using training samples to test sample ratios of 3:2, 2:1, 3:1 and 5:1, the traditional serial BP neural network algorithm, the PSO-BP neural network algorithm and the parallel PSO-BP neural network algorithm proposed in this study were compared in terms of their classification precision ratios and recall rates. The experimental results are shown in Table 1.

**Table 1:** Comparison of the classification performance of different algorithms under different data scales and data partition ratios.

Dataset	Classification algorithm	Accuracy (%)	Data partition ratio				Max	Min	Mean	Standard deviation
			3:2	2:1	3:1	5:1				
Data1										
	BP									
		Precision ratio	84.5	87.6	90.4	92.6	92.6	84.5	88.8	3.04
		Recall rate	89.6	92.2	94.7	95.1	95.1	89.6	92.9	2.21
	PSO-BP									
		Precision ratio	91.9	93.4	95.1	96.3	96.3	91.9	94.2	1.67
		Recall rate	93.8	95.4	97.0	98.5	98.5	93.8	96.2	1.76
	Parallel PSO-BP									
		Precision ratio	95.2	95.9	96.6	97.1	97.1	95.2	96.2	0.72
		Recall rate	97.2	98.0	98.7	99.3	99.3	97.2	98.3	0.78
Data2										
	BP									
		Precision ratio	81.5	84.4	87.2	89.8	89.8	81.5	85.7	3.10
		Recall rate	84.2	87.0	89.7	92.3	92.3	84.2	88.3	3.02
	PSO-BP									
		Precision ratio	90.3	92.0	93.6	94.0	94.0	90.3	92.5	1.46
		Recall rate	91.0	92.8	94.5	96.0	96.0	91.0	93.6	1.87
	Parallel PSO-BP									
		Precision ratio	93.4	95.2	96.0	96.7	96.7	93.4	95.3	1.23
		Recall rate	96.4	97.1	97.9	98.5	98.5	96.4	97.5	0.79
Data3										
	BP									
		Precision ratio	76.2	79.3	82.3	85.2	85.2	76.2	80.8	3.35
		Recall rate	78.7	82.0	84.9	87.9	87.9	78.7	83.4	3.41
	PSO-BP									
		Precision ratio	84.1	86.4	88.5	90.3	90.3	84.1	87.3	2.32
		Recall rate	86.3	88.5	90.7	92.6	92.6	86.3	89.5	2.36
	Parallel PSO-BP									
		Precision ratio	92.2	93.3	94.3	95.2	95.2	92.2	93.8	1.12
		Recall rate	94.1	95.3	96.3	97.1	97.1	94.1	95.7	1.12
Data4										
	BP									
		Precision ratio	69.6	73.2	76.7	80.1	80.1	69.6	74.9	3.91
		Recall rate	74.2	77.4	80.2	83.0	83.0	74.2	78.7	3.27
	PSO-BP									
		Precision ratio	79.0	81.3	83.5	85.6	85.6	79.0	82.4	2.46
		Recall rate	81.9	84.1	86.2	88.0	88.0	81.9	85.1	2.28
	Parallel PSO-BP									
		Precision ratio	90.0	91.3	92.3	93.5	93.5	90.0	91.8	1.29
		Recall rate	91.0	92.3	94.4	95.5	95.5	91.0	93.3	1.76
Data5										
	BP									
		Precision ratio	59.5	63.9	68.1	72.2	72.2	59.5	65.9	4.73
		Recall rate	62.0	66.8	71.1	75.4	75.4	62.0	68.8	4.98
	PSO-BP									
		Precision ratio	68.9	72.3	75.5	78.4	78.4	68.9	73.8	3.55
		Recall rate	70.2	74.5	77.4	80.1	80.1	70.2	75.6	3.67
	Parallel PSO-BP									
		Precision ratio	88.0	89.4	90.5	91.8	91.8	88.0	89.9	1.40
		Recall rate	90.1	91.3	92.5	93.8	93.8	90.1	91.9	1.38

As shown in Table 1, under different data scales and data partition ratios, the classification effect of the algorithm proposed in this study is preferable to the algorithm of the traditional BP neural network and the PSO-BP neural network, showing that the accuracy improvement did not occur by chance. Furthermore, because the efficiency of the traditional BP and PSO-BP algorithms is dramatically reduced as the sample data size increases rapidly [44], the parallel PSO-BP algorithm exhibits the fewest sample fluctuations according to the statistical standard deviation results and achieves the best classification performance. In addition, as the data scale increases, although the classification accuracy rates of all algorithms decreased, the parallel PSO-BP neural network algorithm decreased only slightly, which indicates that the parallel programming model based on MapReduce achieves an excellent performance level, particularly with big data.

#### Speedup ratio and efficiency

**Fig 4:**
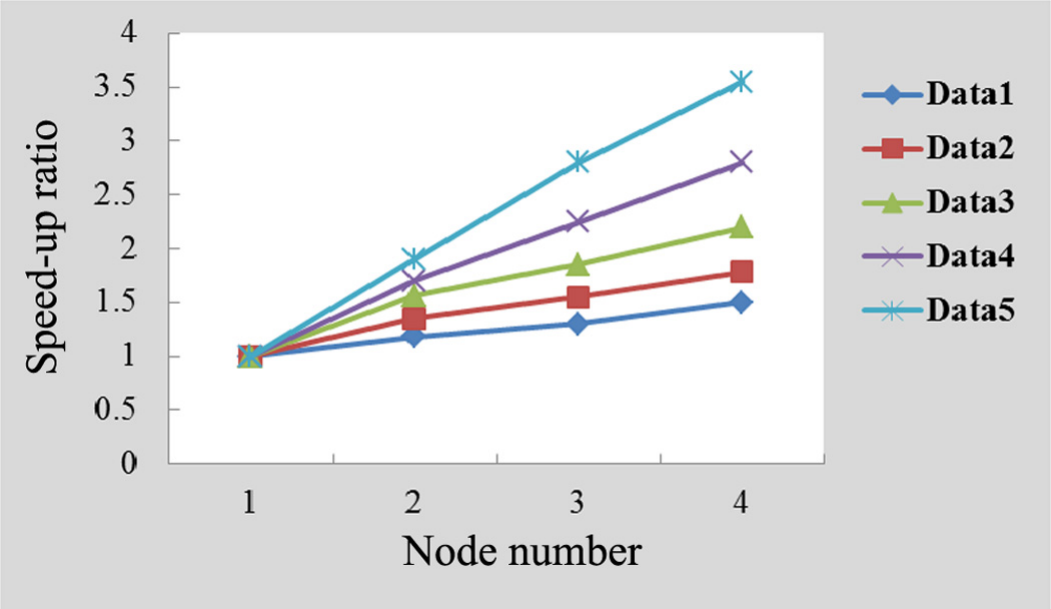
Comparison of speedup ratios with varying numbers of nodes.

**Fig 5:**
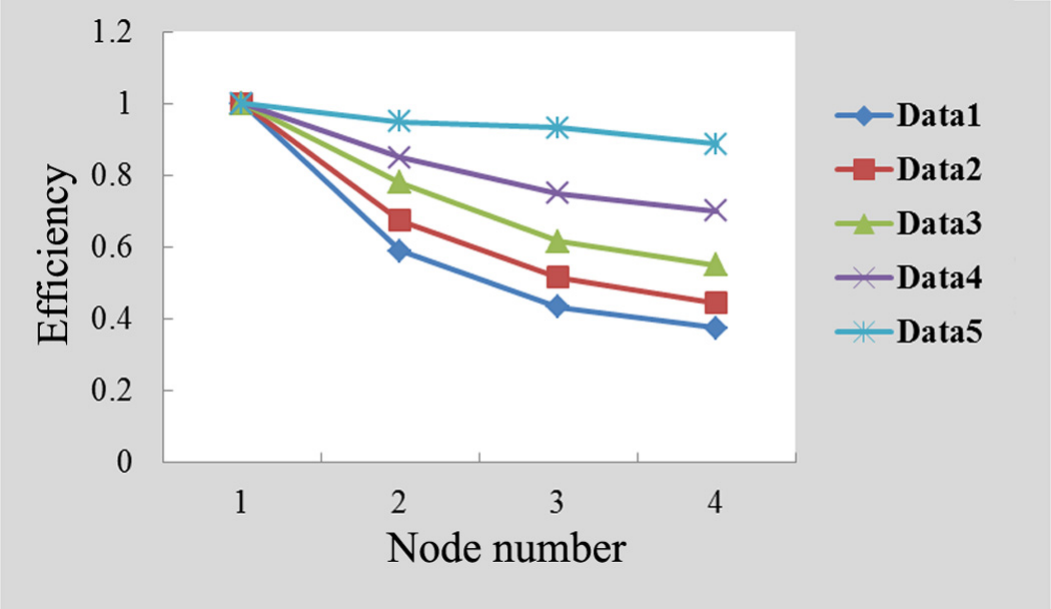
Comparison of system efficiency with varying numbers of nodes.

For the parallel programming model based on MapReduce, two important indicators for measuring algorithmic performance are the speedup ratio and efficiency. The former refers to the ratio of the time required to run a task on a single calculating node to the time required to run that same task on multiple calculating nodes, while the latter refers to the ratio of the speedup to the number of calculating nodes [48]. Under ideal conditions, the speedup ratio increases linearly as the number of calculating nodes increases, and the efficiency remains constant at 1. However, because conditions can be affected by load balancing, communication overhead and other factors, the ratio does not increase linearly, and it is impossible for the efficiency to reach 1. Nevertheless, the study shows that when the efficiency reached 0.5, the system obtained very good performance [48]. Figs 4 and 5 present experimental comparisons of the speedup ratios and of the efficiencies of the algorithm proposed in this study, respectively, using datasets of different scales.

For the proposed algorithm in this paper, the training samples are assigned to a few processors from different slave nodes of the Hadoop cluster, and each processor trains the same BP neural network optimized by PSO and updates the network by counting all the training results. Consequently, on larger datasets, the algorithm's speedup ratio and efficiency performance are improved with increasing number of nodes [45].

In Fig 4, the speedup ratio follows an increasing trend as the number of calculating nodes increases, and increased data size results in an increased magnitude of the speedup ratio. For the same dataset, the processing speed of the system improves as the number of computing nodes increases. In other words, the system consumes less processing time; therefore, the speedup ratio follows an increasing trend. For the different datasets, the multi-computing nodes' performance increases with increasing amount of data [49]. Compared to a single-node computer, the processing speed is much higher. This result further indicates that larger datasets better demonstrate the performance of multiple calculating nodes.

Fig 5 validates the parallel system’s efficiency. Its efficiency on smaller datasets is lower than its efficiency on larger datasets. As the number of calculating nodes increases, the system efficiency decreases more rapidly than when the dataset is smaller. As the size of the dataset gradually increases, the increase in calculating nodes results in a reduced system efficiency although with a smaller magnitude. The main reason for the reduction in system efficiency is that the increase in data size causes the system processing time to increase. In contrast, as the number of calculating nodes increases, the communication overhead between nodes also increases. However, the system efficiency is always greater than 0.5, which indicates that the algorithm has excellent parallel performance and expandability.

In addition, to validate the effectiveness of the proposed algorithm, this study performed image classification prediction on 10,000 randomly selected images from the SUN Database using the proposed algorithm and the algorithm presented in reference [25]. From an accuracy point of view, the average precision ratio and recall rate for the proposed algorithm reached 90.9% and 92.3%, respectively, when there were 4 slave nodes in the Hadoop cluster; however, when using the algorithm presented in [25], the average precision ratio and recall rate reached only 77.8% and 80.2%, respectively. Compared with the approach in [25], the average prediction precision ratio and recall rate based on our method increased by 13.1% and 12.1%, respectively. From the perspective of time overhead, the speedup ratio of the system reached 3.2 for the configuration of the Hadoop cluster in this paper. In other words, for the dataset including the randomly selected 10,000 images, the time cost of the proposed algorithm in [25] is 3.2 times that of our proposed algorithm. Moreover, the time efficiency of our approach has been greatly improved. These experimental results are presumably the result of the following. On the one hand, from a hardware perspective, this study adopted multiple computers to constitute a master-slave mode under a network environment and adopted a multiprocessor, parallel computing strategy to greatly increase the efficiency. On the other hand, from a software technology perspective, this paper uses not only the MapReduce parallel programming framework but also distributed data processing techniques. When the amount of processed data becomes very large, the running time of the traditional algorithm proposed in [25] would be excessive, and the classification accuracy would decrease sharply. These big data conditions reflect the superiority of the proposed algorithm in this paper even more clearly.

## Conclusions

Image classification is a complicated and time-consuming process. It requires more space and time to select, extract, and express features and to use the BP neural network to establish a classification model and classify images. In particular, when the image database size increases sharply, a single-machine environment cannot satisfy the time and space requirements of image classification. As an open-source and distributed computing platform, Hadoop has been widely used because it is convenient and cheap to create clusters and has a simple and easy-to-use computing model. The academic world and the industrial world are continuously studying how to adapt the traditional algorithms and applications developed under single-machine or mainframe environments to a Hadoop cluster environment.

This study conducted an in-depth exploration and analysis of the parallel design and implementation of a PSO-BP neural network algorithm. The study investigated the following three topics: optimizing the initial weights and thresholds for the BP neural network using the PSO algorithm, the PSO algorithm itself, and a parallel design and implementation of the BP neural network. The completed implementation was tested using image data from the SUN Database scene image library. The results verified the performance of the algorithm based on several aspects. The experimental results show that the algorithm proposed in this study achieves a good parallelization that can fully use distributed system resources and improve the algorithm’s classification effectiveness. Furthermore, the distributed parallel system based on MapReduce greatly improved the performance compared with the serial version of the framework and fully demonstrates the powerful calculating capacity of parallel processing.

With the development of parallel technology, parallel computing plays an increasingly important role in addressing the complex problems involved in performing enormous amounts of calculations. The purpose of this study was to apply the MapReduce parallel programming framework to the BP neural network optimized by the PSO algorithm to improve the training speed of the BP neural network by creating a version of the algorithm that runs upon the parallel processing technology of Hadoop clusters. In the field of digital image analysis, using the powerful data processing ability of parallel computing to mine and analyze massive data is helpful for obtaining more accurate image information. This technique is important for image annotation, classification and retrieval and is of great significance in improving machine intelligence in understanding digital images.

As big data and cloud computing continue their rapid development, the processing and analysis of big data will remain a research hotspot for quite some time. The future goals of this study include (1) changing the number of nodes in the Hadoop distributed platform and adjusting related parameters to further improve the algorithm’s efficiency; (2) improving the PSO algorithm to find the global optimal solution in a simpler and faster manner; and (3) optimizing the design of the Map and Reduce tasks for the algorithm to improve its classification accuracy.
